# Problematic and Harmful Social Media Use among Adolescents Receiving Intensive Psychiatric Care

**DOI:** 10.3390/ijerph21101328

**Published:** 2024-10-08

**Authors:** Sarah E. Domoff, Stacey B. Armstrong, Heide Rollings, Amy Mancuso, Mary B. Pacheco, Russell Fridson, Carol A. Janney

**Affiliations:** 1 Department of Psychology, University at Albany, State University of New York, Albany, NY 12222, USA; 2 College of Social Work, The Ohio State University, Columbus, OH 43210, USA; armstrong.1014@osu.edu; 3 Pine Rest Christian Mental Health Services, Grand Rapids, MI 49548, USA; heide.rollings@pinerest.org (H.R.); amy.mancuso@pinerest.org (A.M.); cjanney@umich.edu (C.A.J.); 4 Division of Psychiatry & Behavioral Medicine, College of Human Medicine, Michigan State University, Grand Rapids, MI 49503, USA; 5 Department of Psychology, Russell Sage College, Troy, NY 12180, USA; bernam3@sage.edu; 6 Central Michigan University College of Medicine, Detroit, MI 48201, USA; rfridson@dmc.org; 7 Department of Epidemiology and Biostatistics, College of Human Medicine, Michigan State University, East Lansing, MI 48824, USA; 8 Center for Statistical Consultation and Research (CSCAR), University of Michigan, Ann Arbor, MI 48109, USA

**Keywords:** social media, cybervictimization, cyberbullying, sextortion, adolescent, mental health, problematic media

## Abstract

Although research has shown both positive and negative mental health correlates of social media use, few studies focus on adolescents who are receiving intensive psychiatric care. The purpose of this study was to describe problematic media use, experiences of cybervictimization and sextortion, and correlates with adolescents’ health in a sample of adolescents (*N* = 97; 53.6% female) in a partial psychiatric hospitalization program. Approximately one-quarter of participants reported being cybervictimized at least once over the past month and 17.5% of participants reported ever experiencing sextortion. Greater problematic media use was associated with lower physical activity and greater feelings of loneliness. In a subsample of 51 participants, questions were asked regarding who they had gone to for support regarding experiences of online harm, and barriers to disclosing such experiences. The majority of youth who experienced cybervictimization indicated going to a friend for support but rarely endorsed telling a mental health clinician about it. Even fewer disclosed their experience of sextortion, with nearly half reporting not telling anyone (44.4%). Given the rates of online harm experienced by youth in acute psychiatric treatment, screening for and conducting brief interventions on problematic or risky social media use is recommended.

## 1. Introduction

Social media has become an integral part of adolescents’ lives, profoundly influencing their social development and daily interactions [1]. The ubiquity of social media platforms such as Instagram, Snapchat, and TikTok has transformed how adolescents communicate, form relationships, and perceive themselves [2]. While social media can offer various benefits, including enhanced communication skills, social support, and opportunities for self-expression [3,4], it also poses significant risks, particularly for adolescents who are already vulnerable due to mental health issues [5].

Previous research with non-clinical samples has highlighted both positive and negative mental health outcomes associated with social media use among adolescents. On the positive side, social media can provide a sense of belonging and community, especially for those who may feel isolated in their offline lives [6]. It can also serve as a platform for seeking and receiving social support, which is crucial during the turbulent adolescent years [7,8]. However, the negative aspects of social media use cannot be overlooked. Excessive use has been linked to increased levels of anxiety, depression, and other mental health issues [5,9,10,11]. The constant exposure to idealized images and lifestyles correlates with feelings of inadequacy and low self-esteem [12,13].

Problematic media use in non-clinical samples, often characterized by excessive and compulsive use of social media [14], has been associated with various negative health outcomes [15]. For instance, adolescents who engage in problematic media use are more likely to experience sleep disturbances, reduced physical activity, and increased feelings of loneliness [16,17]. Other harmful online experiences include cybervictimization, such as cyberbullying and online harassment, which is another critical issue faced by adolescents [18]. Research has shown that victims of cyberbullying are at a higher risk for developing mental health problems such as depression, anxiety, and suicidal ideation [19,20,21,22,23]. It is believed that these online events are particularly damaging because they are often anonymous and they can occur at any time, reaching a wide audience [24]. Finally, sextortion, a form of online sexual exploitation where individuals are coerced into providing sexual images or content under the threat of exposure [25], is another significant concern. Adolescents who experience sextortion often suffer from severe psychological distress, including depression, anxiety, stress symptoms, and suicidal thoughts [25,26].

Adolescents often hesitate to report problematic media use due to worry, embarrassment, and a sense of helplessness. They may worry about losing access to their devices or the internet or getting into trouble if they report it [27]. Feelings of embarrassment or guilt, particularly in cases involving sextortion, can lead to a reluctance to disclose the experience, as they may feel responsible or fear judgment [28]. Additionally, adolescents believe that reporting will not lead to meaningful intervention or will make things worse, distrust adults’ ability to handle the situation effectively, and fear a lack of understanding or appropriate action [29].

Despite the growing body of literature on social media use and adolescent mental health, there is a notable gap regarding the experiences of adolescents receiving intensive psychiatric care and their perspectives on seeking support from others, including therapists. These individuals may be at higher risk for problematic media use and online victimization due to their pre-existing mental health conditions, including ADHD (particularly relevant for problematic media use [30]) and internalizing symptoms, such as depression, anxiety, and suicidality (particularly relevant to experiences of online victimization [23,31]). Issues such as problematic media use, cybervictimization, and sextortion are particularly concerning for adolescents in psychiatric care, as they may exacerbate existing mental health problems and hinder recovery.

The current study is descriptive in nature and aims to address this gap by examining problematic media use, experiences of cybervictimization and sextortion, and their correlations with physical activity and loneliness among adolescents in a partial psychiatric hospitalization program. Given that this is a descriptive study, there were no pre-determined hypotheses. We included variables that were of clinical concern during the pandemic, particularly loneliness (related to isolation), and potentially displaced behaviors, such as physical activity. By examining the prevalence and correlates of problematic media use, cybervictimization, and sextortion, this research aims to shed light on the unique challenges faced by these at-risk adolescents in the digital age. Additionally, the study explores the support-seeking behaviors of adolescents who experience online harm and the barriers they face in disclosing such experiences. This is a crucial youth subgroup to understand as the findings may have important implications for clinical practice and highlight the need for targeted interventions to address the impact of social media on adolescents already involved in high levels of care.

## 2. Materials and Methods

### 2.1. Participants

From March 2022 to February 2024, 97 adolescents ages 13–18 completed online surveys (in English; the survey duration was approximately 20 min; recruitment ended when a sufficient sample size was reached for this descriptive study) during the admission process in an adolescent partial hospitalization program (PHP) at a large psychiatric hospital in the Midwest US. All youth admitted to PHP during that time frame were invited to participate in this study. The PHP is a secure and intensive behavioral health treatment program where adolescents are admitted and receive individual and group programming from a multidisciplinary team (psychiatry, social work, nursing, pediatrician, occupational therapist, activity therapist) from 8:00 a.m. until 3:30 p.m., when they return home with a guardian to reinforce the skills that they obtain throughout the day. The average length of stay in the program is 5–7 days, and the program is often a transitional program for individuals who have had inpatient psychiatric hospitalization or are unable to function well in less-intense or community settings such as standard outpatient treatment.

Participants were recruited from the PHP, where they were receiving intensive treatment for moderate to severe behavioral health symptoms, including suicidal ideation, school avoidance, depression, anxiety, disruptive behaviors, and aggression, and who are experiencing functional impairments from symptoms in their home and school settings. The most common primary psychiatric diagnosis for adolescents presenting to PHP include depressive disorders, anxiety disorders, and attention deficit hyperactivity disorder.

Adolescents and their caregivers provided informed consent to participate. The Institutional Review Board approved the study.

See Table 1 for the demographic characteristics of the sample. The majority of participants were white (78.4%), and about half (54%) identified as female. In a subsample of 51 participants, questions were asked regarding who they had gone to for support regarding experiences of online harm, as well as barriers to disclosing such experiences. These participants were not selected purposively; rather, these questions were added later on in the study to enhance understanding of the PHP patients’ experiences.

### 2.2. Measures

The Problematic Media Use Measure Self-Report (PMUM-SR) is an 11-item self-report measure based on Internet Gaming Disorder (IGD) criteria in the DSM-5. The self-report version corresponds with the parent report short form version [14]. Adolescents identify the frequency of symptoms on a Likert scale from 1 (never) to 5 (always). Some sample items include “I lose sleep due to my screen media use” and “Screen media is all that I seem to think about”. Mean scores were calculated, with higher scores indicating greater problematic media use (PMU; α = 0.86). See Table 2 and Table 3 for the frequencies of item endorsement and total scores.

The Addictive Phone Use (APU) Scale [32] is an nine-item self-report measure used to screen for symptoms of dysregulated smartphone use in adolescents and is also based on criteria for IGD in the DSM-5, but applied to smartphone use. Adolescents identify the frequency of symptoms on a Likert scale from 1 (never) to 5 (always). Some sample items include “Have there been times when all you could think about was using your phone?” and “Have you felt that you should use your phone less, but have been unable to cut back on your phone use?” Mean scores were calculated, with higher scores indicating greater addictive phone use (α = 0.86). See Table 2 and Table 4 for the frequencies of item endorsement and total scores.

Three questions to assess for online victimization and bullying were used [33]. Adolescents were first provided with the definition of cyberbullying: “cyberbullying is when someone repeatedly harasses, mistreats, or makes fun of another person (on purpose to hurt them) online or while using cell phones or other electronic devices” [33]. Then, participants were asked how often they experienced cyberbullying (and a separate question on how often they cyberbullied others) in the last 30 days. Responses were provided on a Likert scale from “never” to “many times”. To assess experiences of sextortion, participants were asked “Has someone threatened to expose a sexual image of you to make you do something or for other reasons such as revenge or humiliation?”, providing responses on the same Likert scale from “never” to “many times” [34].

The UCLA Three-Item Loneliness Scale [35] is a self-report screener to assess social isolation and loneliness and measures three domains of loneliness, including relational connectedness, social connectedness and self-perceived isolation. The three items include “How often do you feel that you lack companionship?” How often do you feel left out?” and “How often do you feel isolated from others?” Item responses include “hardly ever,” “some of the time,” and “often,” with higher scores indicating greater feelings of loneliness (α = 0.76).

The Stanford Leisure-Time Activity Categorial Item (L-CAT) [36] is a self-report measure that includes a single item to assess physical activity. The item has adolescents respond to the following statement: “during the past month, which statement best describes the kinds of physical activity you usually did? Do not include the time you spent working at a job. Please read all six statements before selecting one”. Respondents choose from six statements describing activity categories. Category sample items include “almost daily, that is five or more times a week, I did vigorous activities such as running or riding hard on a bike for 30 min or more each time,” and “I did not do much physical activity. I mostly did things like watching television, reading, playing cards, or playing computer games”. Higher scores indicated greater physical activity.

Open-ended questions were added to the original survey after the study began to understand adolescents’ perspectives on their experiences with online victimization. These questions included “has a therapist, counselor, or doctor ever asked you about your social media use? If yes, which treatment provider asked you?” and “Has a therapist, counselor, or doctor asked you about experiencing cyber-bullying? If yes, what did they recommend? Was it helpful or not helpful and why?” Questions also assessed who youth would go to for support if they experienced online victimization and they could identify any of the following: friend, sibling, parent, other family member, teacher, counselor or therapist, an online friend, online support, other (with a write in response option), or “I did not tell anyone”. Questions to assess barriers to disclosing online victimization were also included, such as “Why would you or other teens not want to talk about being cyber-victimized with an adult/someone else?”, and adolescents could choose from as many responses that applied, including embarrassed, fearful it would make the problem worse, concerned that my phone or social media would be taken away, concerned I would get in trouble, and other (with a write-in response option).

### 2.3. Statistical Analyses

Given that this is a descriptive paper, means, standard deviations, and frequencies were calculated on major study variables. Bivariate correlations (using Spearman correlation analysis for non-normally distributed variables) were calculated for the variables of interest. Frequencies of cyberbullying, cybervictimization, and sextortion were calculated, as well as the endorsement of sources of support for these experiences and barriers to disclosing.

## 3. Results

All participants (*n* = 97) were asked about experiences of cybervictimization, cyberbullying others, and sextortion (see Table 5). Twenty-five (25.8%) participants reported being cybervictimized at least once over the past month. Twelve (12.4%) participants reported cyberbullying others at least once over the past month. Seventeen (17.5%) participants reported ever experiencing sextortion.

Spearman correlations were conducted to examine the relationship between study measures, physical activity, and negative online experiences (see Table 5). Greater addictive phone use and problematic media use were associated with greater feelings of loneliness. Similarly, greater addictive phone use and problematic media use were associated with a lower level of physical activity.

Regarding cybervictimization, participants experiencing more frequent cybervictimization over the past month also reported higher amounts of addictive phone use, problematic media use, greater frequency of cyberbullying others, and loneliness. Similar findings emerged with cyberbullying frequency. A greater frequency of lifetime sextortion was associated with greater addictive phone use, problematic media use, and the frequency of past-month cybervictimization and cyberbullying.

A subsample of the total *N* (*n* = 51) was asked about sources of support if they endorsed experiencing cybervictimization in the previous 30 days (*n* = 15; see Table 6). Adolescents most frequently reported that they spoke about the experience to friends (73.3%), followed by an online friend (33.3%), a parent (26.7%), and a sibling (20.0%). Three individuals (20.0%) reported that they did not tell anyone about the experience, and two individuals (13.3%) reported telling a counselor or therapist. One person endorsed telling another family member that was not a parent or sibling (6.7%), one person reported that they got support online (6.7%), and no one endorsed talking to a teacher about it. Of the subsample (*n* = 51), nine participants endorsed experiencing sextortion and were asked about sources of support. Those individuals most frequently reported talking about the experience to friends (44.4%) and telling no one (44.4%). Participants also endorsed talking to an online friend (33.3%) and a sibling (22.2%). None of the participants who had experienced sextortion reported telling a parent, another family member, a teacher, a counselor or therapist, or getting support online.

A subsample of participants (*n* = 51) was asked who they would talk to if they experienced cybervictimization in the future, and they could endorse multiple options (see Table 7). The most frequently reported source of support was friends (72.5%), followed by a parent (52.9%), a counselor or therapist (45.1%), a sibling (41.2%), an online friend (27.5%), and another family member (13.7%). Other less frequent responses included telling no one (11.8%), talking about the experience to a teacher (3.9%), and getting support online (2.0%). Two participants selected “other” (3.9%), and the response included that they would not tell anyone because they would not be bothered by the bullying. The same subsample (*n* = 51) was asked who they would talk to if they were to experience sextortion in the future, and again they could select multiple responses. The most commonly endorsed source of support was friends (54.9%), followed by a parent (52.9%), a counselor or therapist (41.2%), an online friend (21.6%), a sibling (19.6%), another family member (11.8%), and a teacher (11.8%). Individuals also reported that they would not tell anyone (11.8%) and that they would get support online. Three individuals selected “other” (5.9%), and their responses included that they would tell the police, the perpetrator’s parents, or that they know not to send any sexual images, so they would not be in that situation.

Fifty-one participants were also asked about potential barriers that might dissuade them or their peers from talking about cybervictimization in the future (see Table 8). Participants could select multiple reasons. They most frequently reported that they would be fearful reporting would make the problem worse (68.6%), be concerned their phone or social media would be taken away (62.7%), feel embarrassed (60.8%), and be concerned they would get in trouble (56.9%). Among individuals who were asked to consider future barriers to talking about experiences of sextortion (*n* = 51), individuals reported that they may feel embarrassed (72.5%), feel fearful reporting would make the problem worse (72.5%), be concerned they would get in trouble (68.6%), and be concerned that their phone or social media would be taken away (56.9%). Free responses included that they would be open about the experience or that they would be concerned about judgement from others.

A subsample of participants (*n* = 51) was also asked if a therapist, counselor, or doctor had ever asked about who they talk to online or on social media. Of that subsample, 24.5% (*n* = 12) said they had been asked. Advice those participants considered helpful included education about the dangers of social media, to be cautious about who they are talking to online, and to look at recovery pages, which helped one participant feel less alone. Advice from providers that was not helpful included spending less time online and to only message people the individual knows in real life.

Regarding cyberbullying, participants thought that advice to block and/or report offenders was helpful because it cut off contact with the perpetrator. Regarding sextortion, one participant mentioned receiving helpful education regarding the tactics that offenders may use to trick victims into sending images. Other participants said that advice to tell parents or the police if they experience sextortion was not helpful because they were afraid of getting in trouble themselves. One participant noted that they would be more likely to tell a friend, older sibling, or non-family adult who could help but would not get them in trouble.

## 4. Discussion

The purpose of this study was to describe experiences of cybervictimization and sextortion, problematic media use, and their correlates with adolescents’ health in a clinical sample of youth enrolled in an intensive partial psychiatric program. Additionally, the study explored how these adolescents sought support and evaluated the barriers to asking for help when experiencing cybervictimization and sextortion.

Approximately one-quarter of participants reported experiencing cybervictimization in the previous month. These findings appear to exceed rates measured in nationally representative samples. For example, the rate for past year experience of cybervictimization was around 15% among U.S. high school students in 2019 [37]. Our sample reported on past month experiences, which exceeded the non-clinical sample by nearly 10%. Other research has noted the relevance of online stressors to psychiatric hospitalization among youth (e.g., [38,39]). Our findings underscore this relationship and indicate that acute and intensive hospitalization programs should screen for online victimization at admission. Similarly, given the links between cybervictimization and loneliness evidenced in this study, supporting youth in their recovery from online victimization may also extend to building online and/or offline positive social connections to reduce feelings of loneliness.

Regarding experiencing sextortion, 17.5% indicated a lifetime prevalence in our study. This is markedly higher than non-clinical samples. For example, 5% of 12–17-year-olds in the United States reported ever experiencing sextortion [34]. Although the definition provided to youth in our sample did not include other forms of online sexual abuse, the prevalence of sextortion in our sample mirrors other types of harmful online interactions based on the retrospective recall of online sexual offenses during childhood. Finkelhor et al. (2022) describe prevalence rates of approximately 11% for image-based child sexual abuse (e.g., non-consensual image sharing, threatened sharing) in a sample of US adults reporting on experiences before age 18 [40]. Similar to cybervictimization, sextortion (and other forms of online sexual abuse) may be more common among youth seeking psychiatric care, given the notable impacts such experiences have on psychopathology [41].

In addition to examining cybervictimization and sextortion, our study also explored problematic media use and addictive phone use and correlates. Prior research has identified that addictive phone use correlated with greater internalizing symptoms and suicidal ideation; youth with greater addictive phone use were more likely to report that their reason for psychiatric hospital admission was connected to their digital media use (e.g., phone restriction and negative social media experiences [39]). In our study, we examined other behavioral correlates and had similar findings: youth with greater problematic media use (and addictive phone use) reported feelings of loneliness and lower physical activity. Previous research with adolescents has elucidated similar trends. For instance, Varela et al. (2022) explored the relationship between online victimization and loneliness [42]. They found that cybervictimization was positively correlated with loneliness, which was also associated with increased levels of depression. Additional research investigating the impact of cybervictimization on physical activity revealed that cybervictims had reduced physical activity levels, which may be linked to increased levels of anxiety and social withdrawal [43]—mental health concerns commonly associated with being the target of cyberbullying.

### 4.1. Sources of Support for Cybervictimization and Barriers

Adolescents who experienced cybervictimization were asked to whom they disclosed this experience. They most frequently reported speaking about this experience with friends, followed by an online friend, and then a parent or a sibling. As far as seeking support for cybervictimization in the future, participants most commonly reported seeking support from a friend. However, a greater percentage of participants expressed seeking support from a parent, a counselor or therapist, a sibling, an online friend, and another family member in future incidents of cybervictimization. Regarding why adolescents may not disclose these experiences, many expressed concerns about the negative consequences of doing so, such as being fearful that reporting would make the problem worse, being concerned their phone or social media would be taken away, feeling embarrassed, and being worried about getting into trouble.

Despite the common advice given to youth regarding anti-bullying efforts to disclose cybervictimization to adults [44], a large proportion—90% of 12- to 17-year-olds in one study [27]—reported that they would not disclose these experiences to an adult. However, previous research has suggested that adolescents who report cybervictimization to a family member or adult feel good about their choice [45,46] after the event. These results suggest that adolescents may need coaching and support in seeking social support from trusted adults when they experience adverse online events because they may feel better about their choice if they do so.

Some researchers [47] have identified that adolescents would prefer to self-manage cybervictimization. This finding, along with low rates of adult disclosure, would suggest that interventions to support adolescents in gaining skills to self-manage cybervictimization would be beneficial and needed. Clinicians may find it fruitful to provide adolescents with a variety of options for coping with cyberbullying.

### 4.2. Sources of Support and Barriers to Disclosing Sextortion

Compared to sources of support for cybervictimization, a smaller proportion of participants who experienced sextortion reported disclosing the experience to friends (and other individuals), and a greater proportion reported telling no one about their experience. Participants also endorsed talking to an online friend and a sibling. None of the participants who had experienced sextortion reported telling a parent, other family member, teacher, counselor or therapist, or getting support online.

When asked about who they would disclose to in the future, over half of the participants reported that they would get support from friends and parents, with smaller but sizeable proportions also indicating getting support from a counselor or therapist, an online friend, and other family members. A smaller proportion indicated that they would tell no one or would go online to get support. In general, participants were concerned about repercussions that could fall on the victim if they were to tell someone about sextortion. Participants reported that they may feel embarrassed, feel fearful reporting would make the problem worse, be concerned they would get in trouble, and be concerned that their phone or social media would be taken away. Given these concerns, offering adolescents a variety of ways to get support as a preventative measure, as well as supporting parents in having conversations with their youth about what they would do to keep them safe, is recommended.

### 4.3. Screening and Advice from Clinicians

About a quarter of participants indicated that a therapist, counselor, or doctor asked about their social media use. Advice participants considered helpful included psychoeducation about social media risks and benefits, as well as suggested ways to be safe online and find relevant content for recovery (for their respective clinical concerns). Unhelpful advice examples included spending less time online or only interacting with known individuals. Similar preferences were stated regarding psychoeducation on how to be safe online (e.g., how to block or report offenders), and increasing awareness of risks regarding sextortion. Telling parents or the police if they experience sextortion was perceived as not helpful because they were afraid of getting in trouble.

### 4.4. Study Limitations

There are several limitations of this study that should be considered. First, cross-sectional analyses limit our understanding whether the problematic use of screens precedes loneliness and lower physical activity (or vice versa). Future research should examine longitudinal associations and bidirectionality. Although this study includes a high-risk sample, we have a relatively small sample size, with a smaller subsample of participants responding to questions of reporting and barriers to reporting their experiences. To elaborate, the open-ended questions were responded to by a small sample, with limited text responses. As such, we were unable to effectively conduct a thematic analysis. Expanding the research to larger samples of youth receiving intensive psychiatric care is necessary to better capture prevalence rates and screening and treatment preferences. Further, conducting a rigorous qualitative analysis with PHP and inpatient psychiatric adolescent samples in the future may be indicated.

## 5. Conclusions

The results of our study suggest several clinical implications and areas for future clinical research. First, screening for online victimization, sextortion, and other types of risky and problematic social media use is recommended. However, robust screening protocols must be developed and tested before they can be rolled out to clinicians who interact with adolescents. There is also a significant need for the development of brief interventions that can be used when problematic or harmful use is endorsed, given the prevalence of harmful online interactions. Future research should test these brief interventions for safe and healthy social media use in high-risk samples for whom these experiences may be more prevalent (and relevant to treatment). Further, we must also address this issue from a primary prevention perspective. More specifically, we should be establishing safe online practices and providing psychoeducation about online risks to prevent the downstream mental health concerns that can develop if we do not adequately prepare adolescents for the challenges they may experience online.

## Figures and Tables

**Table 1:** Demographic characteristics table. Sociodemographic characteristics of participants (*N* = 97).

	*n*/*M*	%/*sd*
Gender		
Female	52	53.6
Male	32	33.0
Non-binary	7	7.2
Trans	1	1.0
Prefer to self-describe	4	4.1
Age (years)	14.8	1.3
Sexual orientation		
Heterosexual/straight	49	50.5
Gay	5	5.2
Lesbian	8	8.2
Bisexual	24	24.7
Pansexual	4	4.1
Asexual	2	2.1
Questioning/Unsure	2	2.1
Other	2	2.1
Race		
White	76	78.4
Black	3	3.1
American Indian or Alaska Native	2	2.1
Asian or Pacific Islander	1	1.0
Biracial	7	7.2
Other/Prefer to self-describe	7	7.2
Ethnicity		
Hispanic or Latino(a)	17	17.5
Not Hispanic or Latino(a)	76	78.4

Note. Not all participants reported on their demographic background.

**Table 2:** PMUM and APU score frequencies.

**PMUM Score Frequency**	***n***	**%**
1.00–1.99	32	33.0
2.00–2.99	50	51.5
3.00–3.99	13	13.4
4.00–5.00	2	2.1
**APU Score Frequency**	n*	**%**
1.00–1.99	36	38.3
2.00–2.99	39	41.5
3.00–3.99	13	13.8
4.00–5.00	6	6.4

Note. * There were three missing values from the APU scores. Items are responded to on a scale from 1 (never) to 5 (always), with total scores reflecting mean of 11 items (PMUM) or 9 items (APU).

**Table 3:** PMUM item endorsement frequencies.

PMUM Statement	Never *n* (%)	Rarely *n* (%)	Sometimes *n* (%)	Very Often *n* (%)	Always *n* (%)
I lose sleep due to my screen media use.	12 (12.4)	23 (23.7)	33 (34.0)	22 (22.7)	7 (7.2)
2. I sometimes hide my screen media use from my parents.	33 (34.0)	26 (26.8)	17 (17.5)	16 (16.5)	5 (5.2)
3. Screen media is all that I seem to think about.	23 (23.7)	37 (38.1)	25 (25.8)	11 (11.3)	1 (1.0)
4. My screen media use hurts my friendships.	56 (57.7)	26 (26.8)	14 (14.4)	1 (1.0)	0 (0.0)
5. It is hard for me to stop using screen media.	8 (8.2)	33 (34.0)	39 (40.2)	14 (14.4)	3 (3.1)
6. When I have a bad day, screen media seems to be the only thing that helps me feel better.	8 (8.2)	26 (26.8)	35 (36.1)	19 (19.6)	9 (9.3)
7. I am doing poorly in school (not doing well in classes) because of my screen media use.	41 (42.3)	24 (24.7)	21 (21.6)	11 (11.3)	0 (0.0)
8. The amount of time that I want to use screen media keeps increasing.	33 (34.0)	28 (28.9)	30 (30.9)	5 (5.2)	1 (1.0)
9. Screen media is the only thing that motivates me.	48 (49.5)	26 (26.8)	16 (16.5)	7 (7.2)	0 (0.0)
10. I become frustrated when I cannot use screen media.	22 (22.7)	32 (33.0)	21 (21.6)	18 (18.6)	4 (4.1)
11. My screen media use gets in the way of family activities.	20 (20.6)	33 (34.0)	34 (35.1)	8 (8.2)	2 (2.1)

**Table 4:** APU item endorsement frequencies.

APU Question	Never *n* (%)	Rarely *n* (%)	Sometimes *n* (%)	Often *n* (%)	Very Often *n* (%)
Have there been times when all you could think about was using your phone?	20 (21.3)	41 (43.6)	20 (21.3)	13 (13.8)	0 (0)
2. Have you felt restless or tense when you were unable to use your phone?	28 (29.8)	28 (29.8)	19 (20.2)	12 (12.8)	7 (7.4)
3. Have you felt the need to use your phone for longer amounts of time?	19 (20.2)	28 (29.8)	32 (34.0)	11 (11.7)	4 (4.3)
4. Have you felt that you should use your phone less, but have been unable to cut back on your phone use?	19 (20.2)	22 (23.4)	25 (26.6)	17 (18.1)	11 (11.7)
5. Have you spent less time with friends, or doing other activities, in order to use your phone?	43 (45.7)	29 (30.9)	15 (16.0)	6 (6.4)	1 (1.1)
6. Have you had arguments with others about your phone use, but have continued to use your phone excessively?	33 (35.1)	28 (29.8)	20 (21.3)	11 (11.7)	2 (2.1)
7. Have you tried to hide how much you used your phone from family or friends?	46 (48.9)	23 (24.5)	11 (11.7)	10 (10.6)	4 (4.3)
8. Have you used your phone to forget personal problems or other things that were bothering you?	13 (13.8)	6 (6.4)	36 (38.3)	12 (12.8)	27 (28.7)
9. Have you experienced serious conflicts with family, friends, or partner because of your phone use?	47 (50.5)	22 (23.7)	13 (14.0)	6 (6.5)	5 (5.4)

Note. There were three missing values from the APU scores.

**Table 5:** Spearman correlations among primary study variables. Descriptive statistics and correlations for study variables.

Variable	*n*	*M*	*SD*	1	2	3	4	5	6	7
1. APU	94	2.36	0.80	—						
2. PMUM	97	2.32	0.66	0.79 **	—					
3. Loneliness	93	6.42	1.83	0.43 **	0.38 **	—				
4. Physical Activity	97	2.58	1.44	−0.23 *	−0.22 *	−0.16	—			
5. Cybervictimization	97	1.46	0.87	0.44 **	0.42 **	0.30 **	−0.12	—		
6. Cyberbullying	97	1.22	0.62	0.45 **	0.42 **	0.25 *	−0.23 *	0.55 **	—	
7. Sextortion	97	1.29	0.71	0.30 **	0.30 **	0.17	−0.16	0.43 **	0.34 **	—

* *p* < 0.05. ** *p* < 0.01. Note. APU = Addictive Patterns of Use scale. For the APU scale, participants most frequently endorsed using social media apps (*n* = 50), streaming or viewing videos (*n* = 16), messaging on message or text apps (*n* = 14), and gaming (*n* = 7) on their phones. PMUM = Problematic Media Use Measure; the most frequently endorsed media/devices that were problematic for participants were smartphone/mobile phone use (*n* = 64 participants) and gaming (*n* = 7). Twenty-five (25.8%) participants reported being cybervictimized at least once over the past month. Twelve (12.4%) participants reported cyberbullying others at least once over the past month. Seventeen (17.5%) participants reported ever experiencing sextortion.

**Table 6:** Responses from participants who have experienced cybervictimization and/or sextortion (*n* = 17).

	Cybervictimization *n* = 15		Sextortion *n* = 9	
	When You Had Problems Online, for Example, You Were Being Cyber-Bullied, Who Did You Talk to about It?		When Someone Had Threatened to Expose a Sexual Image of You Online, Who Did You Talk to about It?	
	*n*	%	*n*	%
Friends	11	73.3	4	44.4
Sibling (brother or sister)	3	20.0	2	22.2
Parent	4	26.7	0	0.0
Other family member (not parent or sibling)	1	6.7	0	0.0
Teacher	0	0.0	0	0.0
Counselor or therapist	2	13.3	0	0.0
Online friend	5	33.3	3	33.3
I got support online	1	6.7	0	0.0
Other	0	0.0	0	0.0
I did not tell anyone	3	20.0	4	44.4

Note. A subsample of the total *N* was asked about sources of support if they endorsed experiencing cybervictimization or sextortion (*n* = 51). Of the 51, 17 participants reported any experience of cybervictimization or sextortion (33.3%). Those 17 individuals received these questions and could endorse multiple sources of support. Seven participants reported experiencing both cybervictimization and sextortion.

**Table 7:** Responses regarding imagined future response if the participant experiences cybervictimization and sextortion (*n* = 51).

	Cybervictimization *n* = 51		Sextortion *n* = 51	
	In the Future, if You Experience Problems Online, for Example, You Are Cyber-Bullied, Who Would You Talk to about It?		In the Future, if Someone Threatens to Expose a Sexual Image of You Online, Who Would You Talk to about It?	
	*n*	%	*n*	%
Friends	37	72.5	28	54.9
Sibling (brother or sister)	21	41.2	10	19.6
Parent	27	52.9	27	52.9
Other family member (not parent or sibling)	7	13.7	6	11.8
Teacher	2	3.9	6	11.8
Counselor or therapist	23	45.1	21	41.2
Online friend	14	27.5	11	21.6
I would get support online	1	2.0	3	5.9
Other	2	3.9	3	5.9
I would not tell anyone	6	11.8	6	11.8

Note. A subsample of the total *N* received these questions (*n* = 51) and could endorse multiple sources of support.

**Table 8:** Responses regarding imagined reactions dissuading the participant from reporting experiences of cybervictimization and sextortion (*n* = 51).

	Cybervictimization *n* = 51		Sextortion *n* = 51	
	Why Would You or Other Teens Not Want to Talk about Being Cyber-Victimized with an Adult/Someone Else?		Why Would You or Other Teens Not Want to Talk about Someone Threatening to Expose a Sexual Image of You (or Other Teens) Online?	
	*n*	%	*n*	%
Embarrassed	31	60.8	37	72.5
Fearful that it would make the problem worse	35	68.6	37	72.5
Concerned that my phone/social media would be taken away	32	62.7	29	56.9
Concerned I would get in trouble	29	56.9	35	68.6
Other	3	5.9	4	7.8

Note. A subsample of the total *N* received these questions (*n* = 51) and could endorse multiple reasons. “Other” responses for cybervictimization include that they would be forthcoming about cybervictimization or that they are not being cybervictimized. “Other” responses for sextortion include the following: that they would be open about sextortion or they had a fear of being judged by others.

## Data Availability

The datasets presented in this article are not readily available because of privacy restrictions. Requests to access the datasets should be directed to Heide Rollings.
